# Antioxidant Effects of SGLT2 Inhibitors on Cardiovascular–Kidney–Metabolic (CKM) Syndrome

**DOI:** 10.3390/antiox14060701

**Published:** 2025-06-09

**Authors:** Juan Guerrero-Mauvecin, Natalia Villar-Gómez, Lucia Miño-Izquierdo, Adrián Povo-Retana, Adrian M. Ramos, Gema Ruiz-Hurtado, Maria D. Sanchez-Niño, Alberto Ortiz, Ana B. Sanz

**Affiliations:** 1 Laboratorio de Nefrología Experimental, Instituto de Investigación Sanitaria-Fundacion Jimenez Diaz (IIS-FJD), Universidad Autonoma de Madrid, 28040 Madrid, Spain; juguemau95@gmail.com (J.G.-M.); nataliavillargomez@gmail.com (N.V.-G.); mdsanchez@fjd.es (M.D.S.-N.); 2 Departamento de Enfermedades Metabólicas e Inmunitarias, Instituto de Investigaciones Biomédicas “Sols-Morreale”, 28029 Madrid, Spain; apovo@iib.uam.es; 3 Centro de Investigación Biomédica en Red Enfermedades Cardiovaculares CIBERCV, Instituto de Salud Carlos III, 28029 Madrid, Spain; 4 RICORS2040, Instituto de Salud Carlos III, 28029 Madrid, Spain; 5 Cardiorenal Translational Laboratory, Imas12 Research Institute, Hospital Universitario 12 de Octubre, 28041 Madrid, Spain; gemaruiz@h12o.es; 6 Department of Physiology, School of Medicine, Universidad Autonoma de Madrid, 28029 Madrid, Spain; 7 Department of Pharmacology, Universidad Autonoma de Madrid, 28049 Madrid, Spain; 8 Instituto Reina Sofía en Investigación en Nefrología, 28003 Madrid, Spain; 9 Department of Medicine, Universidad Autonoma de Madrid, 28029 Madrid, Spain

**Keywords:** cardiovascular–kidney–metabolic syndrome, treatment, SGLT2 inhibitors, oxidative stress, antioxidant defenses, chronic kidney disease, acute kidney injury

## Abstract

The cardiovascular–kidney–metabolic (CKM) syndrome was recently conceptualized to provide a holistic framework for diagnosing and treating the full spectrum of key age-associated interrelated conditions: overweight/obesity, type 2 diabetes mellitus, acute and chronic kidney disease, and cardiovascular disease. This conceptualization resulted from epidemiological associations, advances in our understanding of shared and interrelated pathogenic mechanisms, and observations that several drug families improved outcomes in all three components of CKM. Sodium/glucose cotransporter 2 inhibitors (SGLT2i) and GLP-1 receptor agonists (GLP-1 RA) enhance all CKM spectrum components, although their efficacy varies against specific components. However, the modified mechanisms by these drugs beyond glycemic control in CKM syndrome are poorly understood. We now deeply review the available literature regarding the impact of SGLT2i on oxidative stress and antioxidant defenses in preclinical and clinical studies of type 2 diabetes mellitus, acute and chronic kidney disease, cardiovascular disease, and CKM syndrome. Evidence suggests that SGLT2i may have a secondary antioxidant effect that reduces the vicious cycle of tissue injury—inflammation—tissue injury, even in organs distant from the primary injury.

## 1. Cardiovascular–Kidney–Metabolic (CKM) Syndrome

Acute kidney injury (AKI) and chronic kidney disease (CKD) are interrelated conditions, with their burden increasing due to population aging and the increasing prevalence of risk factors, such as overweight/obesity and type 2 diabetes mellitus (T2DM) [1]. Around 850 million people suffer from renal disease and CKD, which is predicted to become the fifth global cause of death by 2050, the third in countries with long life expectancy [2,3]. AKI may progress to chronic CKD or accelerate its progression. CKD is a key risk factor for AKI, and it is common for patients with AKI to eventually develop CKD as a lifelong condition [4,5]. Both are associated with an increased risk of premature death, especially from cardiovascular disease (CVD), as well as heart failure (HF), myocardial infarction, stroke, and arrhythmia [5,6]. More recently, cardiovascular–kidney–metabolic (CKM) syndrome was conceptualized to provide a holistic framework to diagnose and treat the full spectrum of key age-associated interrelated conditions: overweight/obesity, T2DM, CKD, and CVD [7,8]. The American Heart Association (AHA) defined CKM syndrome as a systemic disorder in which there is an interplay among the pathophysiology of metabolic risk factors (overweight/obesity, T2DM), CKD, and CVD, resulting in multiorgan damage and premature mortality. Overweight/obesity is a key driver of T2DM, which in turn is the main cause of CKD and a key driver of CVD. Some anti-diabetic drugs, such as SGLT2 inhibitors (SGLT2i) and GLP-1 receptors agonists (GLP-1 RA), have been shown to contribute to weight loss and to prevent and treat T2DM, CKD, and HF, as well as decrease the risk of other CVD outcomes [9,10,11,12]. Oxidative stress is considered a key driver of the CKM spectrum and a hallmark of aging since it contributes to inflammation and tissue injury, activating a vicious cycle of injury–oxidative stress–injury in primarily injured organs and distant organ tissues, and it can be induced by different triggers [13,14]. In this sense, global oxidative status is associated with clinical scores of cardiovascular risks in patients at different stages of CKD [15,16]. Hyperglycemia increases reactive oxygen species (ROS) production and kidneys affected with CKD are a source of ROS [17,18]. SGLT2i exerts antioxidant effects in diabetic patients and in experimental models of diabetes, but the specific mechanisms are not well understood. SGLT2 is overactivated in diabetic patients, leading to altered glucose metabolism and consequent imbalanced redox state [19], based on which SGLT2i can restore the redox state. Hiowever, in experimental cisplatin-induced AKI without diabetes, empagliflozin improved kidney injury and oxidative stress, which was associated with reduced inflammation, suggesting that the antioxidant effect of SGLT2i occurs beyond glycemic control [20]. We now critically review the CKM protective effects of SGLT2i, with an emphasis on their interaction with oxidative stress. However, we do not discuss the differential impact of diverse SGLT2i since the design of most studies did not allow us to evaluate these potential differences in the same experimental settings in a head-to-head manner. The current consensus is that CKM protection by SGLT2i represents a class effect [5,21,22].

### 1.1. Unmet Needs in the Care of AKI and Its CVD Complications

AKI represents a multisystemic disorder that can cause severe complications, causing over 13 million deaths worldwide [23]. AKI is defined as an abrupt decline in kidney function. diagnostic criteria are regularly updated by the Kidney Disease Improving Global Outcomes (KDIGO) (https://kdigo.org/guidelines/acute-kidney-injury, accessed on 2 June 2025). In the clinic, AKI is currently diagnosed by acute increases in serum creatinine or decreases in urine volume beyond certain thresholds. However, these are still markers of kidney function (the condition was previously termed acute kidney failure). Hence, novel identification of kidney injury-associated markers is urgently needed. Potential kidney injury markers include albuminuria, neutrophil gelatinase-associated lipocalin, kidney injury molecule-1, interleukin-18, liver-fatty acid binding protein (L-FABP), insulin-like growth factor–binding protein 7/tissue inhibitor of metalloproteinases–2 (commercial name Nephrocheck), and others [24]. AKI frequently occurs in critically ill, septic patients (incidence over 60%) [25], and in patients hospitalized for non-kidney-related diseases. Importantly, AKI may be complicated by CKD and cardiac dysfunction. However, neither the molecular mechanisms linking AKI to cardiac dysfunction nor the proper diagnostic criteria for cardiac dysfunction in this setting are well characterized. AKI-dependent cardiac injury can be diagnosed by high-resolution echocardiography and by serum biomarkers of HF, such as the increase in aminoterminal pro-brain natriuretic peptide (NT-proBNP) levels and/or cardiac troponin T. However, while increasing NT-proBNP levels are associated with an increased risk of death, NT-proBNP is cleared by the kidneys, and the decrease in glomerular filtration rate (GFR) itself will result in higher NT-proBNP levels [26].

Regional studies, as in Scotland, showed that patients with AKI were four times more likely to suffer myocardial infarction and stroke than the general population [27]. Similar epidemiological studies in other countries are advancing to establish evidence-based cardioprotective actions to prevent cardiovascular dysfunction and death. Further unmet needs in AKI care include early diagnostic markers that detect kidney injury, as discussed above, before kidney dysfunction is evident, and biomarkers that ensure correct stratification and patient-centered treatment of cardiac damage and dysfunction in AKI. Therefore, tools are needed to focus care on upstream events to identify, prevent, and treat efficaciously early AKI instead of just managing its consequences and complications. Further unmet needs relate to stratification criteria linked to the recovery of kidney function and the need for long-term follow-up of cardiorenal health. In this regard, there is currently no therapy that prevents or treats AKI beyond avoiding nephrotoxins, maintaining kidney perfusion, and replacing kidney function when AKI is severe, nor there are any treatments that address the cardiorenal spectrum associated with AKI.

### 1.2. CKD and CKM Syndrome Definitions

CKD is diagnosed when there are abnormalities in kidney structure or function that persist for at least 3 months and current diagnostic criteria is relatively recent (since 2013) [28]. Risk stratification is based on kidney function (GFR <60 mL/min/1.73m^2^) and urinary albumin/creatinine ratio (UACR >30 mg/g) [5]. CKD increases the risk of adverse outcomes such as kidney failure, AKI, CVD events, and CVD mortality [6], and it is frequently caused by metabolic diseases leading to CKM syndrome. CKM is also progressive, and it is divided into five stages (0–4) (Table 1) [7,14].

As CKM syndrome is divided into stages to prevent its confusion with CKD stages, it is important tostratify CKD risk by using the 2013 KDIGO G (GFR: G1 to G5) and A (albuminuria: A1 to A3) categories, rather than the old 2003 CKD stages 1 to 5 [28].

The physiopathology of CKM syndrome is complex. Among multiple players and mechanisms, it involves nitric oxide and reactive oxygen species imbalance, activation of the renin–angiotensin–aldosterone system (RAAS) and sympathetic nervous system, enhanced generation of glycation end-products, hyperglycemia, insulin resistance, lipotoxicity, endoplasmic reticulum stress, mitochondrial dysfunction, inflammation, among other physiopathological features [14].

CKM syndrome has a very high mortality rate, so it is urgent to design new risk prediction equations that allow its prevention and early diagnosis [29]. A valuable therapeutic approach should address the three components of CKM syndrome: renal, metabolic, and CVD. Both SGLT2i and GLP-1 RA reduce CVD and kidney outcomes by mechanisms beyond glycemic control [30,31]. In fact, there is evidence that SGLT2i prevents both CKD and HF in people with T2DM while GLP-1 RA prevents CKD and CVD (mainly ischemic heart disease) in overweighted/obese or T2DM populations [9,10,32]. A healthy lifestyle, including moderate-intensity exercise, dietary control, and weight loss, is crucial for preventing the progression of CKM syndrome [7]. However, the development of overweight or obesity already signals a failure to optimize lifestyles. This likely underlies the high failure rate of lifestyles measured alone to reverse overweight/obesity, as reversal requires more drastic lifestyle changes than prevention. Social and work-related limitations are often insurmountable barriers to developing healthier lifestyles.

## 2. Mechanism of Action of SGLT2i

SGLT2i includes empagliflozin, canagliflozin, dapagliflozin, sotagliflozin, and ertugliflozin [33]. Empagliflozin and dapagliflozin are approved for the treatment of CKD with or without T2DM. SGLT2i decreases glucose and sodium reabsorption in proximal tubules, thereby increasing glycosuria (i.e., calorie loss), which improves glycemic control in diabetic patients and weight in any patient, and urine sodium excretion, which contributes to lower blood pressure and congestion [34,35]. At around 180 g/day of glucose are filtered by glomeruli and reabsorbed completely by sodium/glucose cotransporters, SGLT2 and SGLT1, in proximal tubules, and this amount of reabsorbed glucose increases when there is hyperglycemia or single nephron hyperfiltration [35,36].

SGLT2 is primarily expressed in the kidneys, while SGLT1 is found in the kidneys, intestines, and heart. SGLT2 reabsorbs 90% of filtered glucose in the S1 segment of proximal tubules, whereas SGLT1 reabsorbs the remaining glucose in more distal segments [35]. This reabsorption is driven by the sodium (Na^+^) gradient, which is maintained by Na^+^/K^+^ ATPase in the basolateral membrane through energy expenditure. Glucose then enters the bloodstream via glucose transporter, type 2 (GLUT2) transporters [34,35].

For the treatment of T2DM, SGLT2i offers several advantages over other anti-diabetic drugs, including a lower risk of hypoglycemia and hyperkalemia, weight loss, reduced blood pressure, improved lipid profiles, and lower uric acid levels [33]. Additionally, SGLT2i prevents and treats CKD and HF, and they are also indicated to treat these conditions in the presence or absence of T2DM [33,37,38]. Moreover, protection from severe AKI has been observed in clinical trials [39].

**Figure 1:**
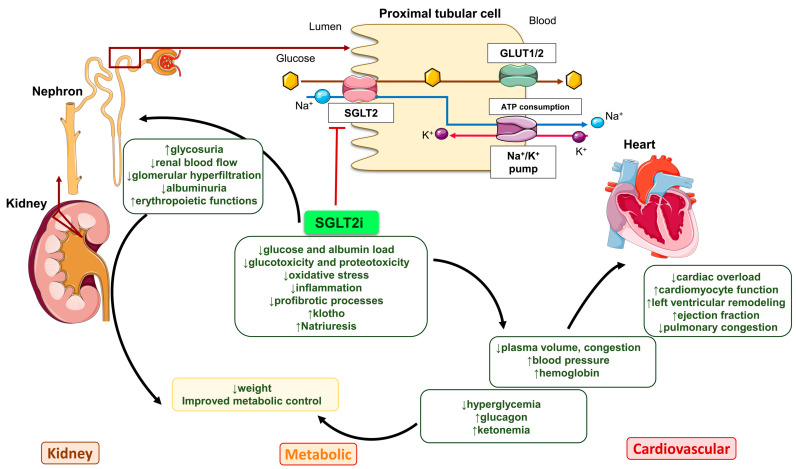
Potential mechanisms of the cardiorenal protective actions of SGLT2i in the CKM syndrome context. The inhibition of SGLT2 in proximal tubular cells prevents glucose entry into these cells, limiting glucotoxicity and potentially reducing oxidative stress, inflammation, and fibrosis, and promoting the expression of the anti-aging and cardioprotective factor Klotho. It also inhibited the reabsorption of sodium (Na^+^), promoting natriuresis that leads to reduced renal blood flow, glomerular hyperfiltration, and albuminuria in kidneys and reduced plasma volume congestion and blood pressure in the vascular system. Decreased renal blood flow also contributes to decreased cardiac overload and increased erythropoietin production in the kidneys, leading to an increase in hemoglobin concentration. At metabolic levels, SGLT2i reduces hyperglycemia, resulting in an increased glucagon level, which contributes to ketonemia, a fuel used by the heart. All of this reduces cardiac overload, facilitating cardiomyocyte function and improving left ventricular remodeling and ejection fraction, potentially increasing myocardial efficiency. Abbreviations: SGLT2i: sodium/glucose cotransporter 2 inhibitors; GLUT1/2: Glucose transporter 1/2; ATP: adenosine triphosphate.

In the kidneys, SGLT2i reduces glomerular hyperfiltration, albuminuria, and the solute and albumin load of proximal tubule cells, protecting them from glucotoxicity and proteotoxicity. This slows the loss of GFR and preserves key non-glomerular kidney functions, such as production of the gerosuppressor Klotho and erythropoietic functions [12,34,35,40] (Figure 1). Increased hemoglobin values favor myocyte oxygenation, while Klotho preservation prevents the negative consequences of Klotho deficiency, which reproduces the cardiovascular phenotype of CKD: cardiovascular calcification, hyperaldosteronism, left ventricular hypertrophy, and myocardial fibrosis [41,42,43,44,45]. SGLT2i-induced natriuresis, lower blood pressure, higher hemoglobin values, lower renal blood flow (the kidneys receive 20% of cardiac output), and ketonemia reduce cardiac overload facilitating cardiomyocyte function, alleviate pulmonary congestion, and improve left ventricular remodeling and ejection fraction, potentially increasing myocardial efficiency [35,46,47,48,49]. Moreover, SGLT2i has an anti-inflammatory and anti-fibrotic effect, which might be linked to reduced oxidative stress [20,50,51,52].

The antioxidant effect of SGLT2i could be mediated through several converging mechanisms, including the reduction in the activity of NADPH oxidase 4, which is the main contributor to reactive oxygen species synthesis [19,53,54], inhibiting the activation of the NLRP3 inflammasome and, therefore, NLRP3-dependent oxidative stress [54,55]. In addition, SGLT2i favors the upregulation of antioxidant systems such as by increasing superoxide dismutase levels [19] and glutathione peroxidase and catalase [56]. More important is the upregulation of the NRF2 system by SGLT2i, which in addition to reducing oxidative stress, promotes the removal of senescent cells [57,58]. Mitochondrial injury is also related to oxidative stress as, in this line, empagliflozin showed a protective effect over mitochondrial quality control in a murine model of diabetic nephropathy [59]. Moreover, in a model of T2DM in rats, empagliflozin reduced mitochondrial ROS via AMPK signaling [60].

## 3. Antioxidant Effects of SGLT2i in Preclinical Models

Preclinical studies allow a detailed characterization of kidney events that cannot be assessed in clinical studies due to a lack of access to kidney tissue. The cellular and molecular pathways targeted by SGLT2i and their interaction with oxidative stress have been explored in murine, rat, or rabbit models of AKI and CKD both in the context of DM and in animals without DM. In some cases, the kidney–cardiac axis was explored.

### 3.1. CKD and CKM Syndrome

Several studies in murine diabetic kidney disease (DKD) have provided evidence of a renal and cardiovascular protective effect of SGLT2i linked to the regulation of metabolic reprogramming and antioxidant effects. Dapagliflozin improved glycemic control and kidney injury in db/db mice, reducing albuminuria and weakly reducing glomerular size. A transcriptomic study of isolated tubular cells uncovered that oxidative phosphorylation was downregulated in dapagliflozin-treated mice [61]. In addition, kidneys from dapagliflozin-treated db/db mice had higher levels of ATP and milder cortical hypoxia compared to untreated mice, while the effect on medullary hypoxia was less pronounced. This was attributed to decreased oxygen and ATP utilization by proximal tubular cells exposed to SGLT2i [61]. Ferroptosis is a regulated form of cell death that requires iron to occur or be activated, and it is ultimately executed by lipid oxidation [62]. Ferroptosis is regulated by several proteins, including glutathione peroxidase 4 (GPX4) and SLC7A11, which act as antiferroptotic proteins, and ACSL4, which acts as a promoter [62,63,64]. In this regard, dapagliflozin reduced features of ferroptosis in the kidneys of db/db mice and human proximal tubular HK2 cells cultured with high glucose and high fat, and this effect was associated with the inhibition of the hypoxia inducible factor 1 subunit alpha/Heme oxygenase-1 (HIF1a/HO1) axis, resulting in increased renal GPX4 expression, decreased renal transferrin receptor (TFRC) expression, and reduced renal iron levels [50]. Dapaglifozin also inhibited kidney ferroptosis in a murine model of DKD, and the β-hydroxybutyrate-calcium/calmodulin-dependent protein kinase kinase 2 (BHB-CaMKK2) axis seems to play a key role in this protective effect [65]. Moreover, dapaglifozin improved high fat-induced obese cardiac dysfunction and this was associated with ferroptosis inhibition by the upregulation of antiferroptotic genes *Gpx4* and *Slc7a11* and by the downregulation of the proferroptotic gene *Acsl4* [66]. Canagliflozin treatment in db/db mice improved renal function and reduced glomerular injury and interstitial fibrosis [67]. A metabolomic study showed that canagliflozin promotes metabolic reprogramming since it reduces carbohydrate metabolism and increased lipid utilization and the glycine, serine, and threonine pathways. Likewise, canagliflozin also increased AMPK phosphorylation and reduced mTOR activation, which may mediate antioxidant effects [67]. SIRT1/SIRT3 are involved in maintaining mitochondrial function and redox homeostasis in different organs including the cardiovascular system [68]. Dapaglifozin protection of cardiomyocytes undergoing hypoxia was reported to be mediated by the restored expression of *Sirt1* and *Sirt3* and the resulting upregulation of antioxidant proteins Superoxide Dismutase 1 and 2 [69].

In a high-fat diet (HFD)-streptozotocin (STZ)-induced T2DM mouse model, dapagliflozin prevented the activation of downstream effectors of the Hippo pathway yes-associated protein (YAP)/transcriptional coactivator with PDZ-binding motif (TAZ), thereby reducing renal inflammation, oxidative stress and fibrosis. However, treatment with rverteporfin, an inhibitor of YAP, was less effective than dapagliflozin, suggesting that the protective effect of dapagliflozin may be mediated by an additional mechanism [70]. In the same murine model, dapagliflozin treatment reduced kidney hypertrophy, fibrosis, proteinuria, and glomerulosclerosis by reducing local CYP4A-dependent 20-HETE production, which diminished NADPH oxidase activity, ROS production, and inflammation [71]. Overall, this suggests that SGLT2i may have an antioxidant effect in diabetic nephropathy, but further studies are required to characterize whether this is dependent on its anti-hyperglycemic effect and whether this antioxidant effect is also related to cardiovascular protection.

The protective effect of SGLT2i may be additive to that conferred by other drugs, such as RAS blockers, vitamin D analogs, or Glucagon-like peptide-1 receptor agonists (GLP1-RAs); other medications used for T2DM and CKD which mimic the effect of the natural hormone GLP1 in lowering serum glucose levels are also associated with anti-inflammatory and antioxidant properties [72]. The combination of empagliflozin with atrasentan and ramipril in db/db mice increased kidney and cardiovascular protection, further decreasing glomerular hyperfiltration, albuminuria, renal fibrosis, and cardiomyocyte hypertrophy [73]. The combination of empagliflozin, semaglutide (GLP1-RA), and ramipril in uninephrectomized db/db mice was more kidney and cardiac protective compared with either glucose-lowering drugs alone with a RAS blocker [74]. Another study compared treatment with empagliflozin or paricalcitol in HFD-STZ-induced T2DM in mice, observing that empagliflozin showed a superior protective effect than paricalcitol on renal injury, oxidative stress, and inflammation, although combination treatment exhibited better nephroprotection [75].

The effect of SGLT2i has also been tested on non-diabetic CKD animal models. Empagliflozin treatment protected from unilateral ureteral obstruction (UUO) as a specific model of post-renal AKI, decreasing kidney fibrosis, inhibiting TLR4 and NFκB pathways, and increasing *Klotho* expression [76]. In CKD induced by subtotal nephrectomy in rats, dapagliflozin reduced kidney fibrosis but did not prevent renal dysfunction. Surprisingly, and contrary to observations in human clinical trials [77], it increased serum creatinine and albuminuria compared with untreated CKD [78]. This result questions the clinical relevance of this animal model, while supporting the potential existence of kidney protective mechanisms beyond reducing albuminuria. Additionally, dapagliflozin treatment in rat subtotal nephrectomy reduced filling pressure and left ventricle (LV) compliance and cardiac fibrosis, but did not increase cardiac perfusion [78]. Cardiac function protection was improved by combining eplerenone and SGLT2i. In a rat model of hypertension induced by a high salt diet, treatment with canagliflozin reduced central volume pressure and renal medullary pressure, which correlated positively with reduced tubular cast area in the juxtamedullary cortex, LV fibrosis, and cardiac hypertrophy [79]. Furthermore, a transcriptomic study in LV disclosed that canagliflozin reduced the expression of genes associated with LV fibrosis and dysfunction [79].

Overall, there is some preclinical evidence for a protective effect of SGLT2i over oxidative stress in CKD, mainly related to protection from ferroptosis, but most preclinical studies did not address an antioxidant effect of SGLT2i in models of CKD with or without DM.

### 3.2. AKI

Diabetes is associated with an increased risk of infection and AKI [80,81]. LPS-mediated AKI in diabetic mice was attenuated by dapagliflozin and in vitro studies in tubular cells challenged with LPS or LPS+dapagliflozin associated this effect with decreased inflammatory cytokines and ROS together with increased expression of ROS-induced antioxidant systems such as AMPK, NRF2, and HO-1 [51]. Furthermore, in diabetic mice with ischemia–reperfusion (I/R)-induced AKI, dapagliflozin decreased AKI severity, lipid peroxidation, fatty acid oxidation (FAO) decay, and abnormal compensatory glycolysis. Parallel experiments also identified the SIRT3/PGC1α pathway, and hence mitochondrial biogenesis, as a target of dapagliflozin [82].

Additionally, the kidney-protective benefits of SGLT2i were investigated independently of diabetes mellitus (DM). Thus, SGLT2 inhibition prevented AKI induced by various insults as well as the progression from AKI to CKD. In cisplatin-induced AKI in rats, empagliflozin improved kidney function and decreased kidney injury severity scores, inflammation, and tissue oxidative stress, as indicated by lower levels of malondialdehyde (MDA) and by preventing the decline in levels of the antioxidant indicators catalase, GPX, and SOD [20]. Dapagliflozin prevented AKI induced by contrast medium in rats, decreasing cell death and preserving renal histology and function. The HIF-1a/HE4/NF-kB pathway was identified as a target of dapagliflozin [83]. Dapagliflozin also prevented AKI induced in mice by aristolochic acid and decreased DNA damage, senescence, and inflammation. In addition, it decreased albuminuria and fibrosis at later stages of AKI progression [84].

In murine I/R-induced AKI, luseogliflozin prevented early and AKI-to-CKD progression, reducing both profibrotic marker expression and extracellular matrix tissue deposition. It also prevented the decrease in SIRT3 expression, mitochondrial biogenesis, and FAO, which is associated with fatty acid accumulation. Luseogliflozin also inhibited ROS generation, lipid peroxidation, and NRF2 and GPX4 loss [52].

SGLT2i has also been tested in preclinical CVD models with renal involvement. Among them, canagliflozin and empagliflozin pretreatment of rats before inducing myocardial infarction prevented the expression of both the early markers of tubular damage KIM1 and NGAL and of the injury mediators NOX2 and NOX4 [85,86]. Furthermore, in a rabbit model of cardiac surgery, dapagliflozin protected against AKI. It was also involved in reducing serum creatinine and urea levels, ROS production, cytokine synthesis, and the upregulation of NRF2-dependent antioxidant enzymes, increasing the urinary input, and improving tubular injury [87].

Overall, there is more preclinical evidence for a protective effect of SGLT2i over oxidative stress in preclinical AKI than in preclinical CKD. This may be related to the most severe impact of AKI on oxidative stress and inflammation, which facilitates the observation of the impact of interventions.

## 4. Antioxidant Effects of SGLT2i in Clinical Studies

Randomized clinical trials have clearly demonstrated the benefits of SGLT2i across the CKM spectrum, as evidenced by guidelines and regulatory approval for the treatment of HF, CKD, or T2DM, with the aim of preventing CKD progression and CVD events and death in the latter two [5,88,89,90]. However, the lack of access to cardiac and kidney tissue makes it more difficult to address their impact on oxidative stress in patients, as almost any experimental approximation is focused on the systemic level rather than the specific tissue level.

### 4.1. CKM Syndrome

Obesity-induced oxidative stress contributes to insulin resistance and T2DM [14]. Hyperglycemia triggers mitochondrial superoxide production, leading to increased oxidative stress and subsequent advanced glycation end-product (AGE) formation, which in turn contribute to tissue damage and inflammation. Additionally, oxidative stress exacerbates endothelial dysfunction, a key factor in diabetic complications. The activation of the local kidney and myocardial RAAS, linked to hyperglycemia, further promotes organ damage by inducing vasoconstriction and fibrosis, thereby aggravating the cardiorenal outcomes [91,92,93,94,95].

Evidence of oxidative stress has been observed in the progression of CKD in patients with DKD, where a correlation between serum oxidized low-density lipoprotein (oxLDL), a marker of oxidative stress recommended by European Food Safety Authority (EFSA) [96], and eGFR was observed [97]. Higher serum oxLDL was also observed in patients with kidney failure and correlated with endothelial dysfunction and higher atherosclerosis risk [98]. Moreover, there is a clear association between oxLDL and NT-proBNP levels even in relatively young subjects (30–50 years) with or without stable coronary artery disease. In fact, oxLDL and NT-proBNP were significantly higher in subjects at high cardiovascular risk analyzed as lifetime risk than in subjects at low cardiovascular risk and this association was independent of traditional cardiovascular risk factors [99]. MDA, another biomarker of oxidative stress recommended by EFSA as support measures [96], is found in serum LDL proteins in diabetic patients [100]. Plasma levels of asymmetrical dimethylarginine (ADMA) in advanced CKD patients have also been associated with poorer cardiovascular outcomes due to imbalanced nitric oxide (NO) production and impaired vasodilation [101]. In addition, products of DNA oxidative damage, also recommended by EFSA [96], such as urinary 8-oxo-7,8-dihydro-2′-deoxyguanosine (8-oxodG), correlates with progression of CKD [102] and serum 8-hydroxydeoxyguanosine (8-OHdG) correlates with CKD mortality rate [103].

Several reports have demonstrated that treatment with antioxidant agents reduces oxidative stress, inflammation, and fibrosis and improves kidney function in diabetic nephropathy patients [104,105], suggesting that drugs with antioxidant effects may have a beneficial effect on CKM syndrome. In this line, SGLT2i has demonstrated significant antioxidant effects in clinical trials involving patients with various diseases, particularly T2DM and CVD and CKD, highlighting their beneficial effects beyond glycemic control. The DEFENCE open-label trial (Dapagliflozin Effectiveness on Vascular Endothelial Function and Glycemic Control) evaluated the effects of dapagliflozin on endothelial function and oxidative stress in T2DM patients. Dapagliflozin treatment improved endothelial function, particularly in patients with higher HbA1c levels, and significantly reduced urinary 8-OHdG levels [106]. In addition, in an observational prospective study of 15 patients with T2DM, empagliflozin treatment reduced systemic inflammation, which was associated with increased glutathione (GSH) levels and antioxidant enzyme expression (GSH reductase and catalase) in leukocytes, which may further contribute to its cardiovascular protective effects [107]. In Japanese patients with congestive HF and T2DM, canagliflozin decreased systemic inflammation and the oxidative stress markers oxLDL [108]. Additionally, a 2-day treatment with dapagliflozin in T2DM reduced oxidative stress, as assessed by a decrease in urinary isoprostanes, independent of blood pressure changes [109]. Also, a recent study on plasma metabolomics in patients with T2DM with non-alcoholic fatty liver disease receiving dapagliflozin was linked to kidney transcriptomics of a different set of patients with DKD (not on SGLT2i), concluding that SGLT2i may protect the kidneys by modifying molecular pathways related to energy metabolism, mitochondrial function, and endothelial function [110].

Several ongoing studies aim to assess the glycemia-independent effects of SLGT2i in CKM. SGLT2i can improve insulin sensitivity [111,112], reduce serum triglycerides [113], and promote a metabolic switch from carbohydrate to lipid usage, potentially reducing fat deposition [114]. These effects may contribute to the overall antioxidant and cardiorenal benefits of SGLT2i in CKM.

The long-term effects of SGLT2i on the risk of new onset CKD were evaluated in a primary care real-world setting in patients with T2DM, observing that these drugs reduced the incidence of new onset CKD as did GLP1-RA [115], supporting the feasibility of primary prevention of CKD in the primary care setting by prescribing glucose-lowering drugs fit for this purpose. In this line, dual treatment with GLP1-RA liraglutide and with the SGLT2i empagliflozin significantly improved serum levels of antioxidant and oxidative stress biomarkers compared with individual treatments. However, when used alone, liraglutide showed a higher antioxidant effect than SGLT2i empagliflozin [116]. Accordingly, an ongoing clinical trial will analyze the cardiovascular effect of GLP1-RAs, SGLT2i, and their combination in T2DM patients, and oxidative stress markers will be measured (NCT03878706). Another clinical trial will analyze the efficacy of the addition of metformin to SGLT2i in diabetic patients with preserved ejection fraction, and oxidative stress will also be measured (NCT06080802).

### 4.2. CKD Without T2DM

The beneficial effects of SGLT2i on CKD of any cause have been well characterized (except for autosomal dominant polycystic kidney disease, as these patients were excluded from the trials) [77,117]. The EMPA-KINDEY study showed that two years of empagliflozin treatment reduced the risk of CKD progression and death from cardiovascular causes in a cohort of patients with a wide range of eGFR and UACR values, and this protection was maintained for at least two years after stopping the trial [117,118].

Indeed, current guidelines recommend SGLT2i as first-line therapy for CKD. Additionally, the effect of SGLT2i on old age patients with HF and CKD was evaluated in the OSHO-heart study. In this study, patients were divided into three groups based on their conventional treatment, tolvaptan, and SGLT2i (empagliflozin or dapagliflozin), with the SGLT2i group showing a reduced risk of HF hospitalization and cardiac death and a better preservation of renal function compared to conventional treatment or tolvaptan treatment, irrespective of diabetes [119]. SGLT2i has also potential therapeutic effects in hereditary podocytopathies. An observational case series study of six patients with hereditary podocytopathies, Alport syndrome, and FSGS showed that the addition of SGLT2i to RAAS blockade was well tolerated and effective in terms of initial eGFR decline and albuminuria reduction [120]. There are currently four clinical trials evaluating the effect of SGLT2i in patients with Alport syndrome (NCT06226896, NCT02378805, NCT06499948, NCT05944016), although none of them will measure oxidative stress.

However, no strong evidence has emerged in patients without T2DM on a systemic or local antioxidant effect of SGLT2i. In patients with CKD or HF without T2DM, SGLT2i decreased inflammation and serum uric acid, as already shown for populations with T2DM [121,122,123]. As oxidative stress may cause and may be caused by inflammation, some authors have proposed that this is consistent with decreased oxidative stress. Hyperuricemia and oxidative stress have also been linked. However, at the plasma pH of 7.4, urate is present in the form of uric acid and behaves as an antioxidant [124]. Therefore, no firm conclusions can yet be drawn about the relationship between SGLT2i and oxidative stress in patients with CKD without T2DM. This clearly represents a research need, especially because CKD progression in low-risk patients enrolled in CKD trials was slower than the spontaneous, age-related loss of kidney function, suggesting that SGLT2i prevents kidney aging [125].

### 4.3. AKI and HF

AKI may trigger cardiac dysfunction. A meta-analysis concluded that AKI increased the risk of developing congestive HF by 60% of ischemic events, such as myocardial infarction, by around 40%, and of CVD mortality risk by 86% [126]. The increased risk of HF is directly correlated to the severity of AKI and the presence of previous ischemic disease. Additionally, one of the most significant risk factors for AKI is a history of HF or a previous myocardial infarction, especially in AKI patients with sepsis [127,128]. Moreover, the increased risk of major adverse cardiac events (myocardial infarction, ischemic stroke, peripheral artery disease) persists for longer than one month after AKI [129]. However, the Assessment, Serial Evaluation, and Subsequent Sequelae of Acute Kidney Injury (ASSESS-AKI) study showed that adjustment for eGFR rate or serum creatinine importantly attenuated the association of AKI with major CVD events (HF, death), suggesting that pre-existing CKD after AKI contributes to its longer-term CVD risk [130].

Mechanistically, several factors are involved in AKI-induced cardiac dysfunction including increased volume overload, acid-base and hydroelectrolyte imbalance, activation of the renin–angiotensin–aldosterone system (RAAS), and accumulation of uremic toxins such as indoxyl sulfate or fibroblast growth factor 23 (FGF-23), which can cause direct myocardial damage [131,132,133]. Some of these factors may be driven by the loss of non-glomerular functions. Thus, tubular functions contribute to volume maintenance, as well as acid-base and electrolyte homeostasis. Moreover, tubules express in the cell surface and secrete Klotho. Klotho is a co-receptor for FGF-23, as well suppressing aldosterone synthesis [42] and having anti-inflammatory, anti-fibrotic, and anti-aging actions. Loss of klotho increases levels of fibroblast growth factor 23 (FGF23). Therapeutic maneuvers that increase klotho expression may reduce FGF23 levels. For example, administration of GDF15 increased klotho expression and reduced FGF23 levels in an experimental model of acute kidney injury (AKI) through folic acid [134]. According to this, the dysfunction of cardiomyocyte contractility and arrhythmias observed in folic acid-AKI were blocked in transgenic mice overexpressing Klotho [132].

Excessive oxidative stress and a shift to an anaerobic energy mechanism with increased glycolytic flux results in a generalized immunological and inflammatory response, which are also relevant mechanisms of AKI-induced distant HF. In this context, the activation of the immune system is involved in both the injury and repair of renal tissue during AKI, and in distant organ dysfunction, such as in HF [135]. Although there are insufficient clinical studies on the impact of SGLT2i on oxidative stress in the context of AKI, some data have emerged for HF. In 80 patients with T2DM and HF, empagliflozin increased the antioxidant capacity by elevating serum activity of SOD and GPx, while reducing MDA levels [136]. In 150 elderly patients with T2DM and heart failure, SGLT2i improved oxidative stress biomarkers such as serum Nox-2 and 8-Isoprostane [137].

## 5. Summary and Future Perspectives

**Figure 2:**
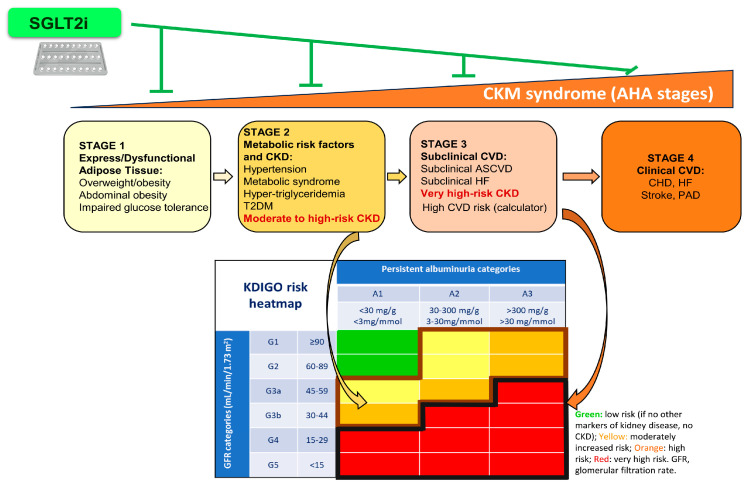
The relationship among AHA CKM stages 1 through 4, KDIGO risk categories G1 through G5 and A1 through A3, and SGLT2i. Stages 2 and 3 of the CKM syndrome include KDIGO high-risk (moderate CKD) or very high-risk (severe CKD) CKD, respectively. KDIGO risk refers to increased risk of CKD progression, all-cause and cardiovascular death, acute kidney injury, and other cardiovascular events (heart failure, myocardial infarction, stroke, atrial fibrillation, and peripheral vascular disease). Note that only KDIGO risk categories G3-G5 convey a diagnosis of CKD, while CKD may be present or absent for KDIGO risk categories G1-G2/A1 or G1-G2/A2. There is evidence that treatment with SGLT2i can prevent the progression of CKM syndrome at all stages by preventing or treating kidney, cardiovascular, and metabolic dysfunction. Abbreviations: AHA: American Heart Association. SGLT2i: sodium/glucose cotransporter 2; CKM: cardiovascular–kidney–metabolic. KDIGO: Kidney Disease|Improving Global Outcomes; CKD: chronic kidney disease; T2DM: type 2 diabetes mellitus; CVD: cardiovascular; ASCVD: atherosclerotic cardiovascular disease; HF: heart failure; CHD: congenital heart disease; PAD: peripheral artery disease.

The overwhelming evidence for the metabolic, cardiovascular (especially for HF), and kidney protective impact of SGLT2i has led to recommendations by major scientific bodies for their use as first-line therapies for these conditions within the CKM spectrum, in addition to the treatment of T2DM [5,21,22] (Figure 2) (Table 2).

Furthermore, preclinical evidence suggests that they reduce oxidative stress in individuals with one or various CKM conditions, which is supported by the scarcer clinical data available. However, it is less clear what the position of the antioxidant effects is in the chain of cellular and molecular events leading to clinical benefit, i.e., a primary event or an event secondary to other beneficial effects of SGLT2i on metabolism, cell stress, or inflammation. The fact that there is no medication in clinical use whose main mechanism of action is decreasing oxidative stress argues against a primary role of the antioxidant effect in tissue protection by SGLT2i. However, the antioxidant effect, even if secondary to other primary events, may contribute to interrupting a vicious cycle of tissue injury–inflammation–tissue injury even in other organs distant from the organ with the specific damage. Future research should carefully delineate the position or requirement for antioxidant effects in tissue protection by SGLT2i. This may help identify populations that benefit the most from SGLT2i once more therapeutic options become clinically available, as well as develop novel therapeutic approaches based on a better understanding of the molecular mechanism of tissue protection by SGLT2i.

## Figures and Tables

**Table 1:** CKM stages and definitions.

CKM Syndrome	Definition
Stage 0: No risk factors	Includes individuals without metabolic risk factors, or no evidence of CKD or CVD. Normal BMI and waist circumference. Normoglycemia. Normotension. Focused on preserving cardiovascular health by carrying out a healthy lifestyle (physical exercise, healthy diet).
Stage 1: Excess/dysfunctional adiposity	Excess/dysfunctional adipose tissue characterized by overweight, abdominal obesity, impaired glucose tolerance, or prediabetes.
Stage 2: Metabolic risk factors and CKD	Metabolic risk factors (hypertriglyceridemia, hypertension, metabolic syndrome, diabetes mellitus type 2) and moderate- to high-risk of CKD.
Stage 3: Subclinical CVD in CKM	CKM risk factors, subclinical CVD (subclinical health failure, atherosclerosis), very high risk of CKD, and high predicted CVD risk.
Stage 4: Clinical CVD in CKM	CKM risk factors and clinical CVD (coronary heart disease, heart failure, stroke, peripheral artery disease, atrial fibrillation) subdivided into: (a) Without kidney failure; (b) With kidney failure.

**Table 2:** Preclinical and clinical benefits of SGLT2i.

CKM Component	Preclinical and Clinical Benefits of SGLT2i (* Preclinical-Only Findings)
Metabolic	Decreased risk of hyperglycemia, hyperkalemia, hyperuricemia, hypomagnesemia. Decreased body weight. Increased ketone levels.
Kidney	Decreased intraglomerular pressure, hyperfiltration, albuminuria, tubular oxygen consumption *. Long-term preservation of GFR. Preservation of non-glomerular kidney functions: erythropoietic (erythropoietin production), gerosuppressor (Klotho production). Increased natriuresis, glycosuria (calorie loss). Protection from AKI, slower progression of CKD, and risk of kidney failure.
Cardiac	Decreased blood congestion. Increased hemoglobin levels. Improve left ventricular remodeling and ejection fraction. Increased myocardial efficiency, reduced cardiac overload. Prevention and treatment of heart failure. Decreased cardiovascular mortality.

* Indicates benefits that have only been observed in preclinical studies.
